# Therapeutic Efficacy of Xanomeline on Psychotropic Drug‐Induced Cognitive Dysfunction in Rats

**DOI:** 10.1002/alz70859_100720

**Published:** 2025-12-25

**Authors:** Cheng Peng, Lifeng Zhang, Zonghao Yu, Juan Tan

**Affiliations:** ^1^ WuXi AppTec, ShangHai, ShangHai China

## Abstract

**Background:**

Psychotropic drug abuse, particularly with amphetamine and ketamine, has been shown to induce alterations in dopaminergic innervation, neuronal structure, and cognitive function. Previous studies demonstrated that the M1R/M4R agonist xanomeline could reverse cognitive abnormalities induced by methamphetamine and MK801, suggesting its potential therapeutic value in addressing psychotropic drug abuse. The aim of the present study was to investigate whether xanomeline, as an M1R/M4R agonist, could ameliorate cognitive dysfunction induced by amphetamine and ketamine abuse.

**Method:**

Male Sprague‐Dawley rats (n=50) were randomly divided into five groups to evaluate the therapeutic efficacy of xanomeline on psychotropic drug‐induced cognitive dysfunction. The novel object recognition (NOR) task, a behavioral paradigm used to assess recognition memory in rodents, was employed. The groups included: Vehicle group (n=10), which received saline injection; Amphetamine group (n=10), which received four injections of amphetamine (2 mg/kg, administered every 2 hours, 72 hours before NOR); Ketamine group (n=10), which received a single injection of ketamine (20 mg/kg, subcutaneously, 30 minutes before NOR); Xanomeline plus Amphetamine group (n=10); and Xanomeline plus Ketamine group (n=10), both of which were administered xanomeline (30 mg/kg, intraperitoneally) 30 minutes before each drug administration.

**Result:**

Pre‐administration of xanomeline effectively counteracted the cognitive deficits induced by amphetamine and ketamine in the novel object recognition task.

**Conclusion:**

The findings suggest a functional interaction between the muscarinic cholinergic and dopaminergic/NMDA systems in modulating recognition memory. Muscarinic cholinergic agonists, such as xanomeline, may hold therapeutic potential for treating cognitive impairments associated with psychotropic drug abuse.

